# Pupil Dilation Reflects Task Relevance Prior to Search

**DOI:** 10.5334/joc.12

**Published:** 2018-01-26

**Authors:** Katya Olmos-Solis, Anouk M. van Loon, Christian N. L. Olivers

**Affiliations:** 1 Department of Experimental and Applied Psychology, Vrije Universiteit Amsterdam, NL; 2 Institute of Brain and Behavior Amsterdam, Vrije Universiteit Amsterdam, NL

**Keywords:** pupil response, attentional priority, visual search, template, working memory

## Abstract

When observers search for a specific target, it is assumed that they activate a representation of the task relevant object in visual working memory (VWM). This representation – often referred to as the template – guides attention towards matching visual input. In two experiments we tested whether the pupil response can be used to differentiate stimuli that match the task-relevant template from irrelevant input. Observers memorized a target color to be searched for in a multi-color visual search display, presented after a delay period. In Experiment 1, one color appeared at the start of the trial, which was then automatically the search template. In Experiments 2, two colors were presented, and a retro-cue indicated which of these was relevant for the upcoming search task. Crucially, before the search display appeared, we briefly presented one colored probe stimulus. The probe could match either the relevant-template color, the non-cued color (irrelevant), or be a new color not presented in the trial. We measured the pupil response to the probe as a signature of task relevance. Experiment 1 showed significantly smaller pupil size in response to probes matching the search template than for irrelevant colors. Experiment 2 replicated the template matching effect and allowed us to rule out that it was solely due to repetition priming. Taken together, we show that the pupil responds selectively to participants’ target template prior to search.

## Introduction

Intelligent vision entails prioritizing information relevant for the task at hand while ignoring irrelevant sensory input. This process is most prominent in visual search tasks, in which the relevant target object must be selected among competing distractors. Theories of visual search assume that observers activate a representation of the relevant target object in working memory – referred to as the *attentional set* or *search template* – which in turn biases attention towards matching visual input (Chelazzi et al., 1998; Desimone & Duncan, 1995; Wolfe, 2007). In line with its top-down nature, template activation ought to occur during the delay period – before the actual search takes place – as this would likely optimize performance (e.g., Carlisle & Woodman, 2011; Desimone & Duncan, 1995; Wolfe et al., 2004). While a body of electrophysiological (i.e., EEG) evidence indeed indicates that template-driven attentional biases occur at least a few hundred milliseconds prior to search (Eimer & Kiss, 2008, 2010; Eimer et al., 2009; Lien et al., 2008), most behavioral studies have measured template-based biases either during or at the very end of the search process, through explicit manual responses (Folk, Remington, & Johnston, 1992; Olivers & Eimer, 2011; Schreij et al., 2014; Wu & Remington, 2003), which leaves room for potential confounds derived from preparing a manual response or from the search process itself.

In search for ways to study behaviorally which template is active during the delay period prior to search, we recently turned to subtle oculomotor responses (Olmos-Solis et al., 2017; Van Loon, Olmos-Solis, & Olivers, 2017). In these first studies, we focused on microsaccades, which in previous work have been shown to provide an implicit measure of spatial attention (Engbert & Kliegl, 2003; Laubrock, Engbert, & Kliegl, 2005; Laubrock et al., 2010; Martinez-Conde, Otero-Millan, & Macknik, 2013). We asked participants to memorize a color as the target for an upcoming visual search task that was presented after a brief delay. During the delay period, we introduced a probe stimulus, which could contain a disk matching the color of the search template. The probes were completely task-irrelevant and participants were asked to maintain fixation. Nevertheless, the results showed that more and larger microsaccades were directed towards probes that matched the relevant template color, suggesting that the task-relevant template-was indeed activated prior to search, and that microsaccades provide a viable measure of task-driven, feature-based attention. Moreover, in contrast to reaction time (RT) measures of attentional bias, such microsaccades can be measured without the need of an explicit response.

In the current study we extended our investigation to another implicit ocular measure: the pupillary response. Previous research have linked the pupil response to cognitive processes such as mental effort or arousal (Beatty, 1982; Boersma et al., 1970; Hess & Polt, 1964; Partala & Surakka, 2003), overall task engagement (Gilzenrat et al., 2010; Jepma & Nieuwenhuis, 2011), violation of expectations (Kloosterman et al., 2015; Preuschoff, ‘t Hart, & Einhäuser, 2011), and memory (Gardner, Mo, & Borrego, 1974; Goldinger & Papesh, 2012; Kafkas & Montaldi, 2011; Kahneman & Beatty, 1966; Naber et al., 2013; Otero, Weekes, & Hutton, 2011). Moreover, the pupil also responds selectively when participants attend to a sub-set of features that carry an intrinsically high interest value, or that are physically more salient (Einhäuser, 2017). However, as Einhäuser (2017) also pointed out, few studies have investigated whether the pupil is directly sensitive to the task-relevance of a feature.

A handful of studies provide indirect evidence for the pupil being sensitive to task-relevance. A study by Mathôt, Van Heusden, & Van der Stigchel (2015) used the pupillary light response (PLR) to track attentional orienting towards task-relevant stimuli during the delay period of a forced choice memory task. When eye movements are controlled for, the typical PLR shows bigger pupil size when covertly attending to a dark area compared to a bright area (Binda, Pereverzeva, & Murray, 2013a; Mathôt et al., 2013). Capitalizing on this effect, Mathôt et al. (2015) asked participants to memorize two colors, followed by a cue indicating which one would be tested after a certain delay. During the delay period two colored probes were presented – one on a bright background and the other on a dark background. One of the disks matched the color category of the memorized color, while the other matched the non-cued color. The results showed evidence that the PLR was sensitive to the brightness of the area surrounding the memory-matching color.^1^ However, in this study, the pupil response was not elicited by the relevant feature (i.e., color) as such, but through the brightness manipulation of the background. In another study, by Smallwood et al. (2011), participants were asked to observe a series of black digits in two task conditions. In the choice reaction time (RT) task, one of the digits would be colored, and participants indicated whether that digit was odd or even. This rendered the black digits task-irrelevant, as participants did not have to attend to them to perform the main task. In a working memory version of the task, one of the digits was replaced by a colored question mark. Here, participants had to report the parity of the *preceding* black digit. Thus, this procedure rendered all black digits potentially task-relevant. The pupil responses to the black digits indeed revealed greater dilation in the working memory task than in the choice RT task. However, the working memory task also involved memory encoding and consolidation of the presented item, whereas the choice RT task did not. Moreover, because the working memory task was overall harder, relevance was also confounded with general mental effort. Finally, a study by Wierda et al. (2012) used an attentional blink paradigm in which observers were instructed to report multiple targets embedded within rapidly presented sequences of distractors. They found that the pupil dilated more for successfully detected targets than for undetected targets (see also Willems, Herdzin, & Martens, 2015). However, although targets are obviously task-relevant, they also require a response, and, in the attentional blink task, memory consolidation. Moreover, detected and undetected targets may differ in terms of the cognitive effort they evoke. Thus, the modulation of the pupil may have reflected any of these processes.

In the current study we aimed to test whether the pupil can discriminate the specific feature relevant for the upcoming task from other, task-irrelevant information. To this end, in Experiment 1, participants memorized one color – the template – for an upcoming search display presented after a delay period. Crucially, we inserted a probe in the delay period, which was always task-irrelevant, but which could either match the color of the relevant search template or be of a new color, not presented in the trial. Participants were instructed to ignore the probe and wait for the search display to appear. This procedure allowed us to measure the pupil response to the task-relevance of stimulus features, while avoiding additional processes associated with having to explicitly respond to a stimulus. Specifically, we compared the pupil response to the probe when it matched the relevant template color versus when the color was not relevant. In Experiment 2, participants memorized two colors, while a retro-cue indicated which of the two was the target in the upcoming search display. Thus, after this cue, the non-cued color was irrelevant and could be dropped from memory. Again, we introduced probes into the delay period that could match the relevant template color, match the color of irrelevant non-cued item, or be a new color not presented in the trial. The non-cued color served as a control for repetition effects, as it was presented and to-be-encoded at the start of the trial but was no longer relevant after the cue.

## Methods

### Participants

Twenty volunteers participated in each experiment. Some participants were excluded from the analyses for excessive amounts of blinks (see the Pupillometry section for a detailed description of the exclusion criteria), and one participant was excluded due to an incomplete dataset (finishing one out of ten blocks). This left a sample of 19 participants (13 females, M = 25.4, SD = 3.4 years old) in Experiment 1 and 18 participants (14 females, M = 23.6, SD = 7 years old) in Experiment 2. All participants were recruited at the Vrije Universiteit Amsterdam. They all had normal or corrected-to-normal vision, normal color perception, and gave written informed consent. All the studies protocols were approved by the Scientific and Ethical Review Board of the Faculty of Behavioral and Movement Sciences of the Vrije Universiteit Amsterdam.

### Stimuli, design, and procedure

#### Experiment 1

**Figure 1:**
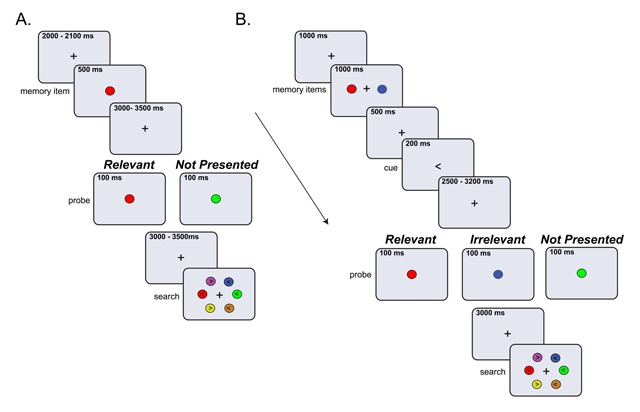
Schematic depiction of a trial in Experiments 1 **A)** and Experiment 2 **B)** Participants memorized one centrally presented color (Experiment 1, A) or two colors (Experiment 2, B) presented to the left and right from fixation. In Experiment 1, the memory item was automatically the search template, target in the search task. In Experiment 2 an arrow retro-cue indicated which color was the target. During the delay period, a probe display was presented, which contained one colored disk. Participants were asked to ignore the probe display and wait for the search display. The task was to search for the memorized color in the search display, and report the orientation (left or right) of the arrow on it. In the example the arrow on the template color (red) points to the left. While in Experiment 1 the search display remained in the screen until response, in Experiment 2, participants had up to 1200 ms to respond.

Figure 1A shows the task design for Experiment 1. Participants pressed the space bar to start the trial, then, a black fixation cross was presented for 2000 to 2100 ms (randomly jittered), followed by the memory display. The memory display consisted of one colored disk (radius: 1.1° of visual angle) located in the middle of the screen which was on for 500 ms. In each trial, the memory color was randomly selected from a set of six (RGB and luminance): red (200,0,0 and 13 cd/m^2^), green (0,140,0 and 14 cd/m^2^), blue (0,90,255 and 15 cd/m^2^), pink (170,68,131 and 14 cd/m^2^), purple (160,40,230 and 16 cd/m^2^), and orange (178,88,0 and 15 cd/m^2^). After a delay in which a fixation cross was shown, we presented the probe display for 100 ms. The probe display contained a colored disk (radius: 1.1°) located in the center of the screen. The experiment had two probe conditions: The Relevant condition in which the probe disk matched the color of the target, and the Not Presented condition where the probe color was randomly chosen from the remaining five colors and therefore it was not previously shown in the trial. To render the presentation of the probe more unpredictable, we varied the delay before the probe with a random temporal jitter between 3000 to 3500 ms. After the offset of the probe, we used another random jitter between 3000 to 3500 ms, which was followed by the search display. The search display comprised six differently colored disks (see above for RGB and luminance values) arranged in an imaginary circle around the central fixation point (radius: 4.6°) with the exact location of each color varied across trials. All disks in the search display contained an arrowhead pointing to the left or right. The direction of the arrowhead for the target and distractors was randomly chosen in each trial. Participants had to locate the disk matching the template color and report the direction of the arrow (left or right) depicted on it, using the arrow keys. The search display remained on the screen until response (there was no time limit), but participants were asked to be as fast and accurate as possible.

All participants completed five practice and 120 experimental trials (8 blocks of 15 trials), with 60 trials per probe condition (Relevant, Not Presented). Therefore, the likelihood of a matching probe was 50%. All probe conditions occurred equally often within a block, but the order of the conditions was fully randomized. Participants received feedback on their performance (RTs and accuracy) after completing each block. Stimulus presentation was controlled with Open sesame version 2.9.7 (Mathôt, Schreij, & Theeuwes, 2012) and presented on a HP Compaq E8400 computer (3 GHz) and a Samsung Sync Master 2233 monitor with a screen resolution of 1680 × 1050 pixels (120 Hz refresh rate). The experiment started with a nine-point eye-tracker calibration and validation procedure. In addition, a one-point recalibration (drift correction) was used at the beginning of each block. We instructed participants to maintain fixation until the search display, avoid blinks and ignore the probe display.

#### Experiment 2

Experiment 2 used the same task and stimuli as in Experiment 1, except for the following: First, the memory display contained two colored disks, placed to the left and right from fixation at 2.1° of visual angle. Second, the memory display appeared for 1000 ms and was followed by a fixation cross and then a spatial retro-cue, each presented for 500 ms. This cue indicated which of the two disks was the target for the upcoming search by means of an arrow pointing left or right (0.2° visual angle). The non-cued color could be dropped from memory and was no longer relevant. We had three probe conditions: The Relevant and the Not Presented conditions were the same as in Experiment 1, but we added the Irrelevant condition where the probe matched the non-cued color. In addition, we shortened the durations of the inter stimulus interval (where the fixation cross was shown) to compensate for the extra time needed for the additional probe condition. Therefore, the start of the trial was now temporally jittered between 1000 to 1500 ms, while the fixation before the probe was jittered between 2500 to 3200 ms, and the delay after the probe was fixed at 3000 ms. As in Experiment 1, participants had to locate the disk matching the template color and report the direction of the arrow (left or right) depicted on it, using the arrow keys. Moreover, to induce a strong representation of the target color, we limited the response time to a maximum of 1200 ms.

All participants completed 180 experimental trials (10 blocks), preceded by six practice trials. There were in total 60 trials per probe condition (Relevant, Irrelevant and Not Presented). Therefore, the likelihood of having a matching probe was 33%. All probe conditions occurred equally often within a block, but the order of the conditions was fully randomized. Each color was used equally often as the search template. We provided similar instructions as in Experiment 1.

### Pupillometry

Participants sat in a dimly lit room, 70 cm away from the computer screen with their heads placed on a chin rest to restrict head movements. The left eye’s horizontal and vertical positions as well as the pupil area were monitored with an Eyelink 1000 desktop mount (SR Research Mississauga, Canada), a video-based eye tracker with 1000 Hz temporal and 0.2° spatial resolution.

Preprocessing and further analysis were done in Matlab (Mathworks). We performed linear interpolation of the blinks automatically detected by the Eyelink software and those additionally detected using peak detection on the velocity of the pupil signal. When two blinks occurred in close proximity (i.e., at most 4 samples or 250 ms between the end of the first blink and the beginning of the next blink) they were treated as single event. All blink events were replaced through linear interpolation from 100 ms before the starting point until 100 ms after the endpoint of the blink, a procedure based on several earlier studies (Kloosterman et al., 2015; Knapen et al., 2016; Urai, Braun, & Donner, 2017). Both blinks and saccades can have a long lasting effect on the pupil size (up to five seconds in case of blinks). To remove those effects, we created saccades and blinks regressors by convolving all saccades and blinks (per subject) with the standard Impulse Response Function (IRF, Hoeks & Levelt, 1993). We then used these regressors to estimate their effect on the pupil signal in a general linear model (GLM, see: Knapen et al., 2016, for detailed description of the method). The residual pupil time series were bandpass filtered using a 0.01–10 Hz second-order Butterworth filter, z-scored, and resampled to 100 Hz. Next, we segmented the pupil signal into trials time-locked to the probe onset, including 500 ms before the probe and 3000 ms after probe presentation. The average pupil size in the interval from –500 ms to 0 ms to probe onset was used as a baseline. We baseline-corrected each trial by subtracting the mean baseline pupil size from each sample in the trial.

We excluded from the analysis subjects who had an excessive amount of blinks, using the following criteria: If, after down-sampling, more than 40% of the total number of trials contained interpolated samples, the subject was excluded. As a result, we excluded one participant in Experiment 1 and one in Experiment 2. The remaining participants had on average 5.7% (Experiment 1), and 8.8% (Experiment 2) of trials with interpolated samples. To ensure that the correct search template was held in memory, only correct trials were included in the pupil analysis. However, when running the analysis with all trials we obtained similar results.

### Statistical analyses

For manual responses, mean RTs and response accuracies were tested for significant differences across probe conditions, using paired sample t-tests, which in Experiment 2 were preceded by a repeated-measures omnibus ANOVA.

**Figure 2:**
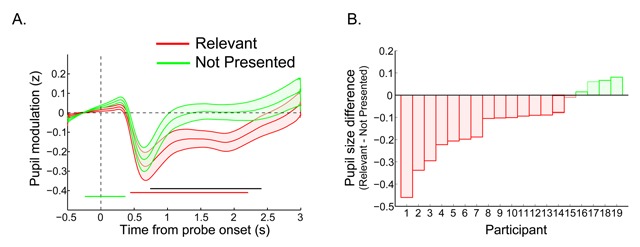
**A)** The time course of the pupil response for Experiment 1: The probe was presented at time zero. The thick lines indicate the mean pupil diameter, and the shaded areas indicate the standard error (SEs) across subjects. Colored (horizontal) bars indicate clusters of significant modulations; black line indicates significant differences between colored traces (*p* < 0.05 cluster corrected, N = 19). **B)** Average pupil effect per participant: A negative value indicates that the pupil was smaller when the probe matched the relevant color. Per subject we calculated the mean pupil size in the interval 740 to 2410 ms in the Relevant condition and subtracted the mean size of the Not Presented condition.

**Figure 3:**
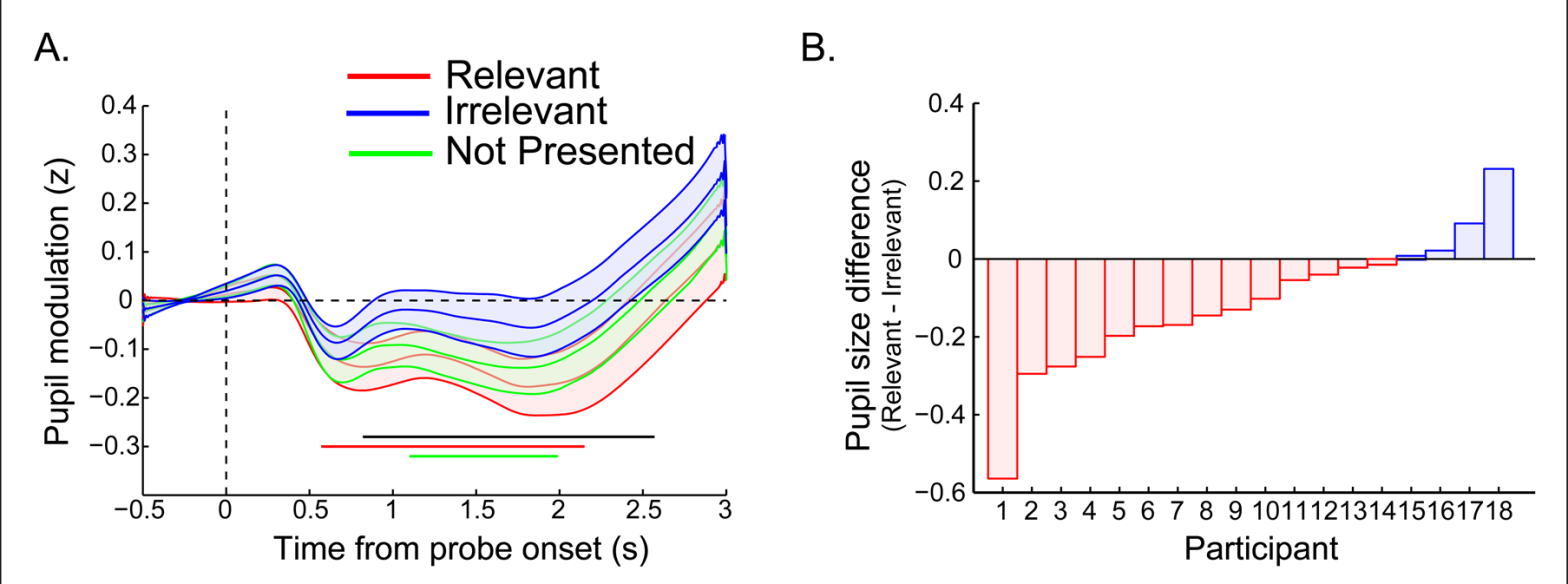
**A)** The time course of the pupil response for Experiment 2: The probe was presented at time zero. The thick lines indicate the mean pupil diameter, and the shaded areas indicate the standard error (SEs) across subjects. Colored (horizontal) bars indicate clusters of significant modulations; black line indicate significant differences between the Relevant and Irrelevant probe conditions (*p* < 0.05 cluster corrected, N = 19). **B)** Average pupil effect per participant: A negative value indicates that the pupil was smaller when the probe matched the relevant color. Per subject we calculated the mean pupil size in the interval 820 to 2570 ms in the Relevant condition and subtracted the mean size of the Irrelevant condition.

As for pupil size, we tested for condition differences in the pupil time series, using group-level permutation testing with cluster correction for multiple comparisons, as implemented in the Fieldtrip toolbox (Maris & Oostenveld, 2007). For each sample, depending on the comparison of interest, we computed *t* values of the difference between two pairs of conditions or between a condition and zero (i.e., the baseline for z-scored data). These *t* values were thresholded at a *p* value of 0.05, yielding clusters of significant differences. Next, labels of trials (from the conditions being compared) were randomly shuffled and a *t* test was performed on the shuffled data. This procedure was repeated 2000 times. We saved the sum of *t* values within the largest cluster into a distribution of summed cluster *t* values. This distribution reflected the null-hypothesis of no difference between conditions. Finally, we summed the observed (nonpermuted) cluster *t* values, and thresholded them using the null-hypothesis distribution (specifically, the percentile that corresponded to the same *p* value as used for the initial *t* test; e.g., the 95th percentile for *p* < 0.05). This approach ensures a correction for multiple comparisons by taking into account clusters of rate modulations that can be expected by chance. In order to provide additional information on the reliability of the findings, we display the size and direction of the effects for each subject, for the time window within which we found significant modulations at the group level (Figures 2B, 3B). More specifically in Experiment 1, for each subject we calculated the mean difference in pupil size of the Relevant minus the Not Presented condition, for the 740–2410 ms time window. For Experiment 2 we did the same for the conditions that showed significant differences, i.e., Relevant- Irrelevant, using the 820 to 2570 ms window.

Finally, we performed an exploratory analysis in which we computed across-trial correlations between the pupil size and reaction times (RTs). For each subject and condition, we calculated a Spearman-rank correlation between the pupil size at each time point and the RTs of the trials from a given probe condition. This resulted in a time course of correlation values per subject and conditions, which were Fisher transformed to ensure the validity of the statistical testing. Next, the resulting correlations time courses were tested for significant differences using group level permutation testing with cluster correction as described above.

## Results

### Experiment 1

#### Search performance

Table 1 shows the mean percentage correct and mean reaction times (RTs) for the search task, split up by the different preceding probe conditions (Relevant and Not Presented). Paired sample *t*-tests revealed that while participants were similarly accurate in both conditions [*t*(18) = 0.95, *p* = .353], they were on average 43 ms faster when the probe disk matched the relevant template color than when it was a new color, not presented in the trial [*t*(18) = 2.7, *p* = .013, *d* = 0.63].

**Table 1:** Percentage correct and RT in the search task as a function of probe condition (N = 19).

	Percentage correct		RT	
	
Probe Condition	Mean (%)	SD	Mean (ms)	SD

Relevant	91.5	2.7	1006	267
Not Presented	90.8	4.4	1059	231

#### Pupil response

Figure 2A shows the time courses of the pupil response for the Relevant and the Not Presented probe conditions. Using a cluster corrected permutation test (see the Statistical analyses section), we tested when the pupil response differed significantly a) from the pre-stimulus baseline, and b) between probe conditions, here Relevant and the Not Presented. As can be seen in Figure 2A, the pupil response to the probe stimuli started with a small dilation, which for the Not Presented condition was significant 240 ms prior to 370 ms after probe onset. After this initial dilation, a pronounced constriction occurred for both conditions, as would be expected given the stimulus-associated change in luminance and contrast (Loewenfeld, 1958). However, while the pupil time course for the Irrelevant condition returned to baseline about one second after probe onset, the time course for the Template condition remained significantly below baseline from 440 ms to 2210 ms after probe onset. A significant probe condition difference appeared from 740 ms after probe onset and extended up to 2410 ms.

To further visualize the reliability of this effect, for each participant we averaged the pupil response over the significant time window (740–2410 ms) and then computed the difference in pupil size of the Relevant minus the Not Presented condition. Figure 2B shows a bar graph where each bar represents the direction and size of the effect for each subject. A negative value (depicted in red) indicates that the participant had a smaller pupil size in response to probes matching the relevant template than for not presented colors. As can be seen in Figure 2B, most subjects (15 out of 19) showed this effect.

Thus, the pupillary response proved sensitive to differences between stimuli matching task-relevant representations and new stimuli, not matching a memory representation.

### Experiment 2

Experiment 1 showed that the pupil is sensitive to the task-relevant search template prior to the search task: The pupil size was smaller in response to probes matching the relevant template color than to probes of a not presented color. However, in Experiment 1, the template-matching probe was a repetition of the memory display. Therefore, the observed difference across conditions could be due to repetition priming, rather than task-relevance as such. Therefore, Experiment 2 aimed to dissociate task relevance from potential repetition effects. We always presented two colored disks in the memory display, after which a retro-cue indicated which color was to become the search template. After the cue, the non-cued color was irrelevant (see Figure 1B). As in Experiment 1, the probe consisted of one single disk presented at fixation, which could match either the relevant template color, the irrelevant non-cued color, or it could be a new color not presented earlier in the trial. Crucially, the Irrelevant condition allowed us to dissociate relevance from repetition, as this color was seen earlier in the trial but was no longer relevant for the upcoming search task. On the basis of the previous experiment we predicted a smaller pupil size in response to probes matching the template color than for those matching the irrelevant color as well as for new colors, not presented in the trial.

#### Search performance

Table 2 shows the mean percentage correct and reaction times (RTs) on the search task. A repeated-measures ANOVA with Probe condition (Relevant, Irrelevant and Not Presented) as within-subjects factor, revealed that the probe condition had an effect on how accurate participants were at reporting the direction of the arrow [F(2,34) = 3.36,p = 0.047,\eta _p^2 = 0.16]. Participants were more accurate in the Relevant than in the Irrelevant [*t*(17) = 2.40, *p* = .028, *d* = 0.57] and Not Presented [*t*(17) = 2.12, *p* = .048, *d* = 0.51] conditions, while the Irrelevant and Not Presented conditions did not differ from each other [*t*(17) = 0.27, *p* = .786]. There was also a main effect of probe condition on RTs [F(2,34) = 5.65,p = 0.008,\eta _p^2 = 0.25]. Participants were, on average, 37 ms faster in the Relevant than in the Irrelevant condition [*t*(17) = 3.76, *p* = .002], while the Relevant vs. Not Presented [*t*(17) = 1.58, *p* = .131] and the Irrelevant vs. Not Presented [*t*(17) = 1.62, *p* = .124] conditions did not differ from each other. Thus, similar to Experiment 1, we observed a benefit in search performance when the probe matched the relevant template color, as participants were both more accurate and faster in the Relevant than in the Irrelevant condition.

**Table 2:** Percentage correct and RT in the search task as a function of probe condition (N = 18).

	Percentage correct		RT	
	
Probe Condition	Mean (%)	SD	Mean (ms)	SD

Relevant	92.9	2.4	722	54
Irrelevant	90.1	4.9	739	52
Not Presented	89.7	5.4	731	62

#### Pupil response

Figure 3A shows the time course of the pupil response to the probe display for the Relevant, Irrelevant and the Not Presented conditions. As in Experiment 1, the probe display led to a pronounced constriction of the pupil which, after cluster correction, was significantly below baseline for the Relevant (from 570 to 2150 ms) and Not Presented conditions (from 1100 to 1990 ms) but not for the Irrelevant condition. Importantly, we observed a relevance effect starting at 820 ms until 2570 ms after probe onset, where the pupil was significantly smaller in response to probes matching the relevant template color than for probes matching the irrelevant color, with the Not Presented condition in between. The Not Presented condition was not significantly different from either the Relevant or the Irrelevant condition.

To further visualize the reliability of this effect, for each participant we averaged the pupil response over the significant time window (820–2570 ms) and then computed the difference in pupil size of the Relevant minus the Irrelevant condition. Figure 3B shows a bar graph where each bar represents the direction and size of the effect for each participant. A negative value (here in red) indicates that on average, the subject had smaller pupil size in response to probes matching the relevant template than for the irrelevant color. As can be seen in Figure 3B, most subjects (14 out of 18) showed this effect.

Summarizing, we confirmed the finding that the pupil is sensitive to the task-relevance of visual input, here expressed as the pupil being smaller in response to visual input matching task-relevant representations compared to previously seen but irrelevant representations. This shows that the effect is not merely driven by repetition, since in both conditions the probe color was presented in the memory display.

### Across-trial pupil size by reaction time correlations in Experiment 1 and 2

**Figure 4:**
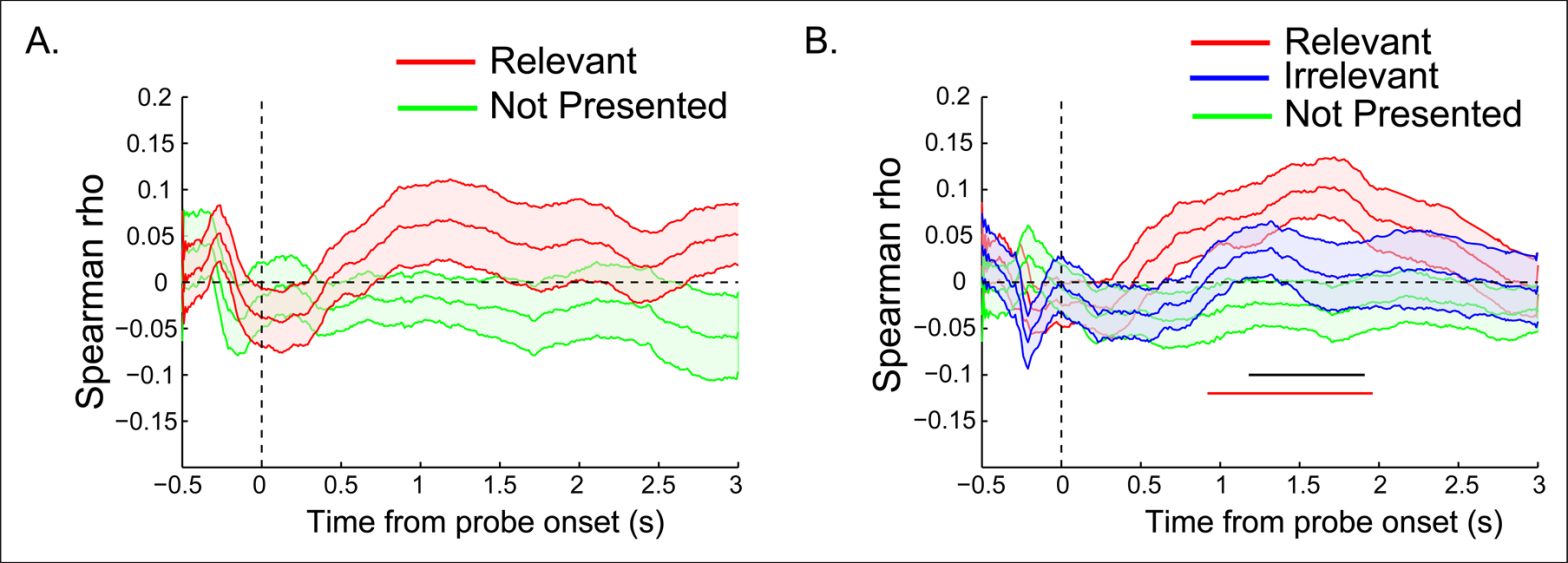
**A)** The time course of RT by pupil response correlation for Experiment 1 and **B)** Experiment 2: The probe was presented at time zero. The thick lines indicate the mean Spearman rank correlation, and the shaded areas indicate the standard error (SEs) across subjects. Colored (horizontal) bars indicate clusters of significant correlation (i.e., rs different from zero). Black bar in B corresponds to the difference between the Relevant and Not Presented condition (*p* < 0.05 cluster corrected).

Finally, we investigated whether the pupil response to the probe display predicts search speed on a trial-by-trial basis. Figure 4A shows the time courses of the average rank-correlations for each condition in Experiment 1, while Figure 4B shows the same for Experiment 2. In both experiments, the Relevant condition showed positive correlation values between pupil size and reaction time (RT), indicating that trials with greater constriction (i.e., smaller pupil size) also showed faster RTs. However, when tested against baseline (zero), only in Experiment 2 these correlations survived cluster-based correction for multiple comparisons, for a time window between 850 ms and 1910 ms from probe onset, while correlations in the Irrelevant and Not Presented conditions did not significantly differ from zero. In the same experiment, for the time window between 1250 and 1850 ms, there was also a reliable condition difference in correlations between the Relevant and the Not Presented probe condition. Thus, the pupil response to a template-matching probe was predictive of how rapidly observers would find the target in the subsequent search display.

Taken together, the pupil exhibits a selective response to the task-relevant search template compared to other Irrelevant or not previously presented visual input. Moreover, the magnitude of this response is linked to subsequent search performance, providing additional evidence that the pupil responds to task relevance.

## Discussion

We investigated whether the pupil response can discriminate which feature is relevant for an upcoming task. To that end, we studied the pupil response to a probe stimulus presented during the delay period, while participants held in memory the task-relevant target feature (i.e., color) and waited for a search task to arrive. Importantly, the probe stimulus itself was task-irrelevant, and thus did not require a response, but it could carry the task-relevant color. Our results demonstrated that, before the actual task takes place, the pupil discriminates between task-relevant and task-irrelevant features, as template-matching probes led to significantly smaller pupil size than probes of new colors, not previously seen in the trial. Moreover, Experiment 2 ruled out that this effect was driven by mere repetition priming, as probes matching the relevant template color also led to more constriction than probes matching a color that was seen in the trial but that was no longer relevant for the task. Therefore, we demonstrated that an implicit ocular measure – the pupil response – discriminates which stimulus is relevant for an upcoming search task, without the need of inducing an explicit, task-related motor response.

Previous studies have reported links between the pupil response and task-relevant stimuli, showing that the pupil dilates more when a target is detected among distractors (Kang & Wheatley, 2015; Privitera et al., 2010; Smallwood et al., 2011; Wierda et al., 2012) and that the extent of such dilations can explain individual differences in performance (Willems, Herdzin, & Martens, 2015). However, compared to those studies our results show that the pupil can signal the task-modulated relevance of a particular feature. Moreover, we evaluated the pupil response to a stimulus that could carry the task-relevant feature, but was effectively irrelevant in the context of the task, as participants did not have to respond to the probe and were instructed to ignore it. This led to some methodological advantages: first, it allowed us to measure the effect of task-modulated relevance on the pupil response, without the confounding effects of the need for additional manual responses, which have a strong effect on the pupil (for an example see: Privitera et al., 2010). Second, because participants did not know in advance whether or not the probe would match the template color, general arousal cannot explain the observed differences. That is, participants could not have been systematically more engaged in one condition than another. Furthermore our results could not be explained by surprise or violation of expectations. In our second experiment, pupil size in the Not Presented condition fell in between the Relevant and the Irrelevant condition, even though only in the Not Presented condition the color was new (and thus potentially most surprising) within the context of a given trial. Instead, we only found significant differences between the Relevant and Irrelevant conditions which both repeated one of the original memory colors with equal likelihood (33%), but differed in terms of the relevance of the color. Therefore, the differential pupil response to the probe matching the template color most likely reflects participants’ task settings for that specific color.

Our results connect with previous neurophysiological evidence indicating that target features are actively represented in the brain during the delay period, prior to the task (Barak, Tsodyks, & Romo, 2010; Ede, Niklaus, & Nobre, 2016; Larocque, Lewis-Peacock, & Postle, 2014; Lewis-Peacock, et al., 2012; Rainer & Miller, 2002; De Vries, Van Driel, & Olivers, 2017) and that the neural representation of upcoming visual input is enhanced, when it matches the template (Eimer & Kiss, 2008, 2010; Eimer et al., 2009; Gayet et al., 2017; Lien et al., 2008; Peters, Roelfsema, Goebel, 2012). Note however that although our data discriminates which memory is task-relevant, it does not directly speak to the presence of match-induced attentional biases during the delay period, since the pupil does not provide a spatial measure of attentional orienting (see Binda et al., 2013; Mathôt et al., 2015 for an indirect, pupil-derived measure of attentional orienting). In this respect, our findings may not be specific to memory for visual search. Nonetheless, our earlier work has shown that the representational enhancement of the relevant memory translates into attentional biases, as expressed in the pattern of microsaccades during maintenance, prior to the task (Van Loon, Olmos-Solis, & Olivers, 2017; Olmos-Solis et al., 2017).

A remaining question is why the pupil constricts rather than dilates in response to the matching probe. As mentioned in the introduction and in contrast to our current study, Wierda et al. (2012) observed dilations in response to relevant target letters in an attentional blink paradigm. However, while in our present study the probe was task-irrelevant and participants did not have to respond to it, in Wierda et al. the stimulus of interest did not only match the relevant feature but was also task relevant in itself, in that it was a target. Moreover, as observers had to memorize the identity of target letters and report it at the end of the trial, the effect is confounded with memory encoding which can also lead to pupil dilations (Goldinger, Papesh, 2012; Hoffing, Seitz, 2015). Any of these factors could explain the differences in the direction of the effect. One possible explanation for the constriction effect found in the current study is that as template matching information is being enhanced, it becomes subjectively more salient than non-matching input, making the stimulus appeared brighter than it actually is. Our effects could then be an indirect measure of task-relevance, mediated by this increase in subjective brightness. Indeed previous studies have shown modulations of the pupillary response by the subjectively perceived brightness of a stimulus. For instance, Binda, Pereverzeva & Murray (2013b) reported that images containing the sun elicited greater pupil constriction than control images without the sun, even though they were paired in terms of their luminance and contrast (See also: Naber & Nakayama, 2013). However, such indirect induced salience effects will be difficult to disentangle from direct top-down relevance effects, exactly because such top-down processes are thought to increase the gain (and therefore behavioral salience) on target-matching objects.

Another possibility is that the constriction effect reflects an effort to avoid guidance by the template-matching feature, when the stimulus carrying this feature (i.e., the probe) is not relevant for the task. Notice that only in the Relevant probe condition, we found a positive correlation between trial RT and pupil size. Although this correlation needs to be taken with caution, it might suggest that participants were faster in the search task, when the pupil response to the probe stimulus was attenuated, i.e., the pupil was smaller or constricted. In this respect, our results are in line with those of Mathôt et al. (2015), who also found evidence from the pupil response that observers tried to avoid intervening probes, as the PLR appeared to reflect the luminance of the side other than the one containing the matching color. This potential ‘inhibition’ of template-matching information during the delay is reminiscent of our previous observation that during the delay period – prior to a search task – microsaccade production was more suppressed in response to template matches. Presumably, participants avoided being captured by matching, but irrelevant probes, as precipitated guidance could hamper performance in the upcoming search task (Olmos-Solis et al., 2017). This is not to say that pupil constrictions to probes of relevant colors reflect the strategic deployment or withholding of attention to the probe display. Instead, we believe the constriction effect was reactive, as a result of the match between the perceptual input and the active template.

Notice that search RT was on average shorter when observers had been presented with the template-matching probe. This could fuel the argument that observers deliberately attended the probe to refresh their memory, and thus saw it as useful “target” stimulus in itself. The probability of having a color repetition in the probe display was high and could potentially lead to the strategic use of the probe (Experiment 1: 50%, Experiment 2: 33%). However, previous studies have shown dilation rather than constriction when people treat an object like a target (Smallwood et al., 2011; Wierda et al., 2012), and the fact that we found constriction speaks against this possibility.

One might also argue that instead of responding to the specific task-relevant feature, the differential pupillary response was actually driven primarily by the *irrelevant* probe colors. Observers may have applied greater effort to suppress probes of irrelevant colors as they might hamper performance on the subsequent search task. As pointed out in the introduction, greater cognitive effort generally leads to pupil dilation and our results may reflect (relatively) greater pupillary dilation when suppression of irrelevant colors was needed. However, there are at least two arguments against this. First, as many previous studies have shown, memory-matching (rather than mismatching) stimuli tend to be the most distracting ones (Kiyonaga, Egner Soto, 2012; Olivers, 2009; Oliver, Meijer, Theeuwes, 2006). Second, if the effects are caused by the way in which mismatching visual input is processed, one would predict correlations between pupil size and RTs in the Irrelevant and Not Presented probe conditions, with more dilation leading to faster RTs. However, only the Relevant condition showed a correlation, as smaller pupil size (i.e., more constriction) led to faster RTs, suggesting that the pupil selectively responded to the task-relevant feature. Regardless, note that even if the pupil selectively responds to task-*irrelevant* features, it would still be sensitive to task relevance overall.

Finally, there is the important question as to what the physiological basis is of a relevance-driven pupillary response. Although a causal relationship is yet to be established, a predominant hypothesis is that the locus coeruleus (LC) drives the cognitive effects observed in the pupil. This hypothesis is supported by the strong correlation between pupil dynamics and LC activity (Joshi et al., 2016; Murphy et al., 2011; Rajkowski, Kubiak, & Aston-Jones, 1993). However, as argued by Einhäuser, (2017) the LC may have limited role in activating task-specific representations, as it has been predominantly linked to general arousal, overall task engagement, surprise, and uncertainty, but much less to attentional orienting or specific priority of important features. Given the reported effects of attentional orienting on the pupil response, recent studies have explored whether other subcortical and cortical brain structures, known to be involved in controlling attentional deployment, also control the pupil size. For example, microstimulation of the frontal eye fields (Lehmann & Corneil, 2016) and the superior colliculus (SC; Wang et al., 2012) can lead to prominent pupil dilations without saccade production. Moreover, a study by Lovejoy and Krauzlis (2010) suggests a causal involvement of the monkey SC on covert spatial attention to goal-related signals, as reversible inactivation of this area led to impaired performance in a selective attention task. Interestingly, inactivation of the SC alters the relationship between covert spatial attention and microsaccades (Hafed, Lovejoy, & Krauzlis, 2013). Therefore, the SC may be a brain structure driving the priority signal as found in microsaccades and potentially in the pupil response. More studies are needed to establish a clear causal link between the activity of cortical and subcortical brain structures and the attentional relevance effects observed in the pupil.

## Data Accessibility Statement

Data from the studies reported in this manuscript are publicly available on the Open Science Framework link: https://osf.io/dmfuv/, DOI: https://doi.org/10.17605/OSF.IO/DMFUV

## Footnotes

Note however, that the effect was opposite to what was expected: The PLR appeared to reflect the luminance of the side other than the one containing the matching color. Observers may have tried to avoid the memory-matching probe, as it may have interfered with the main task. Nevertheless, this still indicates an effect of task-relevance.
